# Oncofertility and COVID-19: At the Crossroads between Two Time-Sensitive Fields

**DOI:** 10.3390/jcm11051221

**Published:** 2022-02-24

**Authors:** Valentin Nicolae Varlas, Roxana Georgiana Borș, Anca Lucia Pop, Bogdana Adriana Năsui, Nicolae Bacalbasa, Roxana Bohîlțea, Radu Vlădăreanu, Corina Manolea

**Affiliations:** 1 Carol Davila University of Medicine and Pharmacy, 8 Eroii Sanitari Blvd., 050474 Bucharest, Romania; valentin.varlas@umfcd.ro (V.N.V.); anca.pop@umfcd.ro (A.L.P.); nicolaebacalbasa@gmail.com (N.B.); vladareanu@gmail.com (R.V.); corina.manolea@drd.umfcd.ro (C.M.); 2 Department of Obstetrics and Gynaecology, Filantropia Clinical Hospital, 011171 Bucharest, Romania; 3 Department of Clinical Laboratory, Food Safety, Carol Davila University of Medicine and Pharmacy, 6 Traian Vuia Street, 020945 Bucharest, Romania; 4 Department of Community Health, Iuliu Hațieganu University of Medicine and Pharmacy, 6 Louis Pasteur Street, 400349 Cluj-Napoca, Romania; adriana.nasui@umfcluj.ro; 5 Department of Obstetrics and Gynaecology, Cantacuzino Clinical Hospital, 030167 Bucharest, Romania; 6 Department of Obstetrics and Gynecology, Elias Clinical Hospital, 17 Mărăști Blvd., 011461 Bucharest, Romania; 7 Assisted Reproduction Department, Columna Medical Center, 021522 Bucharest, Romania

**Keywords:** oncofertility, SARS-CoV-2, COVID-19, young cancer patient, assisted reproductive techniques, cryopreservation, psychosocial, telemedicine

## Abstract

Background: COVID-19 infection has dominated our lives and left its mark on it. The impact on fertility is major, and the long-term consequences may be disastrous. When we talk about oncofertility, we are talking about those patients worried about the delay in receiving medical services (possible cancelation of surgery, decreased availability of medical services, reorientation of medical resources) due to COVID-19. Finally, patients’ worsening biological and reproductive statuses, associated with high levels of anxiety and depression, are closely related to social restrictions, economic impact, reorientation of medical resources, health policies, and fears of SARS-CoV-2 infection. Aim: We reviewed the current literature on fertility during the COVID-19 pandemic and its effect on cancer patients. Specifically, how cancer treatment can affect fertility, the options to maintain fertility potential, and the recovery options available after treatment are increasingly common concerns among cancer patients. Methods: A systematic literature search was conducted using two main central databases (PubMed^®^/MEDLINE, and Web of Science) to identify relevant studies using keywords SARS-CoV-2, COVID-19, oncofertility, young cancer patient, cryopreservation, assisted reproductive techniques (ART), psychosocial, telemedicine. Results: In the present study, 45 papers were included, centered on the six main topics related to COVID-19. Conclusions: Fertility preservation (FP) should not be discontinued, but instead practiced with adjustments to prevent SARS-CoV-2 transmission. The increased risk of SARS-CoV-2 infection in cancer patients requires screening for COVID-19 before FP procedures, among both patients and medical staff in FP clinics, to prevent infection that would rapidly worsen the condition and lead to severe complications.

## 1. Introduction

Oncofertility stands at the crossroads between adult and pediatric oncology and reproductive medicine. Cancer in young patients is reported in those aged 15 to 39 years, and it has unique characteristics due to risk factors, type of cancer, histopathological form, therapy, prognosis, and survival [1]. According to the WHO, approximately 400,000 children aged 0 to 19 years old are diagnosed with cancer each year, with a rate of curability that reaches 80%. In cancer survivors, fertility is a major concern in 1% of patients younger than age 20, and 10% younger than age 45 [2]. As cancer treatments are continuously developing and due to early diagnosis and at younger ages, long-term survival is a greater probability for cancer patients interested in preserving their childbearing ability and achieving parenthood [3,4]. Cancer in patients of reproductive age affects 50–75% of the fertility potential. This proportion can sometimes be much lower depending on the type of malignancy, and the treatment provided [5]. 

Oncofertility’s multidisciplinary approach, of a reproductive medicine specialist in collaboration with an oncologist and gynecologist, focuses on the importance of informing a patient about available procedures for maintaining fertility, balancing the benefits against the risks of treatment delay for each case, monitoring treatment effects, the ability of reproduction after treatment and potential routes of conception. 

**Figure 1:**
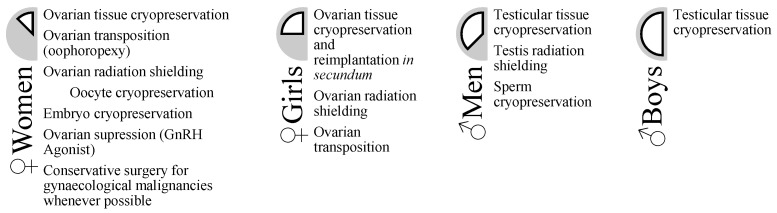
Fertility preservation (FP) approaches to the cancer patient (from Mayo Foundation for Medical Education and Research) [10].

Several strategies are currently established for the fertility preservation of cancer patients [6,7,8,9] (Figure 1). The choice of the best FP strategy is highly dependent on gender, age, marital status, treatment gonadotoxicity (depending on molecules and cumulative doses), ovarian reserve for female patients, patient overall health at cancer diagnosis, psychological status, and amount of time before cancer treatment begins.

The novel coronavirus (COVID-19) was declared a pandemic in March 2020 by the World Health Organization (WHO), and is caused by severe acute respiratory syndrome coronavirus 2 (SARS-CoV-2), with a high rate of infectivity and life-threatening complications, especially in patients with comorbidities [11].

With a limited addressability to FP procedures before therapy initiation, patients undergoing gonadotoxic cancer therapies with or without regard to FP have been further highly fragile during the COVID-19 pandemic since March 2020.

The COVID-19 pandemic reduced the use of health systems by a third due to restrictions and orders to stay at home, which led to an increase in telemedicine. These measures also affected patients diagnosed with cancer by delaying diagnosis and postponing surgery due to the cancelation of services, changing cancer treatment according to quickly published guidelines, and fear of becoming infected during medical visits [12]. This reorganization of health systems has increased the psychological burden on cancer patients and medical staff, preventing them from providing essential care to these patients on time [13]. Although FP is considered an emergency among cancer patients, there has been a reduction in fertility services during this period. Due to the increased risk of infection due to cancer and its treatment affecting the immune system, patients have preferred isolation and given up the desire to procreate [14].

Cancer patients have a 2.3 times higher risk of SARS-CoV-2 infection than the general population due to frequent contact in the hospital environment and immunity issues from disease therapy [15]. Additionally, studies have shown a higher mortality rate, a real need to increase the hospitalization rate, and challenging resource management due to COVID-19 [16,17]. Moreover, in COVID-19 patients, it is unclear how much an added decline in fertility is present due to the virus. There are scarce data about oncofertility status during the pandemic and the influence of COVID-19 on fertility preservation interventions.

The main objective of the present study is to systematically review the recent scientific data on oncofertility during the COVID-19 pandemic.

## 2. Materials and Methods/Data Search

In the present paper, we performed a systematic descriptive review with search compliance with the Preferred Reporting Items for Systematic Review and Meta-Analysis (PRISMA) guidelines 2020 on published papers regarding topics (1) “oncofertility”; (2) “young cancer patient”; (3)”cryopreservation”; (4) “assisted reproductive techniques”; (5) “psychosocial”; and (6) “telemedicine”—with a data filter on “COVID-19”—published in a scholarly peer-reviewed journal and written in English or French (but with no country restriction) between March 2020 and October 2021. The initial review protocol assumed a preliminary Google Scholar search, restraint to two major databases—PubMed^®^/MEDLINE and Web of Science Core Collection—with a preliminary Google Scholar^®^ Database scan.

Inclusion criteria are represented by cancer patients of reproductive age, oncofertility care and interventions, evaluation of oncofertility services, the role of telemedicine in oncofertility, psychosocial impact, and influence of COVID-19 infection on fertility and cancer.

## 3. Results

The PubMed^®^ search retrieved for “oncofertility” 178 results, with 38 reviews, 4 systematic reviews, and 2 clinical trials. Association of “COVID-19” and “oncofertility” revealed 3 results; “oncofertility” and “young cancer patient” retrieved 41 results; “oncofertility” and “assisted reproductive techniques” retrieved 84 results; “oncofertility” and “cryopreservation” retrieved 46 results; “oncofertility’” and “telemedicine” retrieved 1 result; “oncofertility’” and “psychosocial” retrieved 3 results. Search over the WoS database retrieved for “oncofertility” 206 results (120 articles and 38 reviews), showing an emerged health issue and an increased focus on the topic. For the association “oncofertility” and “COVID-19”, the search retrieved 4 results; “oncofertility” and “young cancer patient” retrieved 66 results, “oncofertility” and “assisted reproductive techniques” retrieved 6 results, “oncofertility” and “telemedicine” retrieved 1 result, “oncofertility” and “psychosocial” retrieved 7 results, “oncofertility” and “cryopreservation” retrieved 74 results.

The results revealed a constant interest, but scarce data have been published regarding the fertility preservation of cancer patients during the COVID-19 pandemic.

## 4. Cancer Patients and COVID-19

Cancer patients are susceptible to SARS-CoV-2 infection due to biological status, associated conditions (obesity, diabetes, cardiac, pulmonary, or renal disease), and impaired immune status secondary to cancer and treatment (especially chemotherapy) that may alter the host response to SARS-CoV-2 infection [18,19]. Despite the attempt at self-isolation, the risk of these patient contamination is very high due to increased hospital visits. The severe form of COVID-19 is significantly more probably due to cancer comorbidity with an intense and fatal evolution. This probability is doubled for recent surgery or chemotherapy treatments [20]. Of the total COVID-19 deaths, 20.3% of the patients had an active cancer form [21]. Eight in 100 of all COVID-19 deaths in Romania are associated with cancer. A study regarding the prevalence of comorbidities in 814 COVID-19 deaths pointed to cancer with an 18.4% figure of all associated comorbidities [22].

El Gohary et al., in a meta-analysis on over 1000 cancer patients with confirmed SARS-CoV-2 infection, revealed overall mortality of 21.1% (95% CI: 14.7–27.6) together with an OR of 3.91 and 4.86 for severe/critical disease and mechanical ventilation, respectively when compared to infected non-cancer cases. The analysis concluded that cancer patients are at a higher risk of COVID-19 infection-related complications than non-cancer patients [23], and so all efforts must be made to ensure, as much as possible, a COVID-free environment around these already vulnerable patients.

Cancer services worldwide had to adapt to the COVID-19 pandemic to minimize risk to patients and staff. A reduction in systemic anticancer treatment was registered after the debut of the COVID-19 pandemic [24]. Clark et al. observed a significant reduction in cancer patients’ medical records for subsequent therapy lines at the beginning of the pandemic (32% reduction in April 2020, 10% reduction in May 2020) with a maximum for non-curative indications. Subsequently, there was an increase in May 2020 for curative and adjuvant treatments, immunotherapies, and first-line non-curative therapies. In June 2020, most registrations increased for most tumor types, except for neoadjuvant therapies [24]. This evolution is synchronous with the pandemic peaks, a pattern that was replicated with the post-lockdown or post-pandemic peak periods. Concerning the lockdowns or other limitations, the indication is to prioritize urgent procedures of FP in close connection with the chronology of treatments specific to cancer.

Van de Poll-Franse et al., in a cross-sectional study based on an online questionnaire completed by 4094 patients, revealed changes in cancer care in the first weeks of the COVID-19 crisis in one out of three patients. Thus, to diminish the exposure to SARS-CoV-2, first-line anticancer therapy on younger cancer patients was canceled or postponed, and a follow-up appointment was replaced with telemedicine consultation (telephone or video consultations) [25].

In 6.8% of newly diagnosed patients, delays in cancer management were observed in the first 7 months of 2020 compared to the previous year. Despite this, in previously diagnosed patients, there was a 4% increase rate [26]. In a prospective international cohort study of 20,006 adult cancer patients (≥18 years) in 61 countries, a disruption of surgical management was observed in 0.6% cases for mild restrictions, 5.5% for moderate, and 15% for lockdowns [27].

In a cross-sectional study of 6676 patients, Patel et al. showed that women are better informed than men (56%). Women aged 30 years with a high fertility potential in the future were counseled in 67.7% of cases on impaired fertility associated with chemotherapy and in 55.6% on FP options, then 10.5% of patients who did not were advised about the risk of impaired fertility and the FP options [28].

During the COVID-19 pandemic, cancer-screening programs were deferred, which would mean that incipient cancers were undiagnosed, leading to an increase in cancer morbidity and mortality in the coming years [29,30,31]. Further assessment is needed to evaluate the consequences of providing less or delayed treatment initiation, particularly for newly diagnosed cancers [26].

## 5. What Are the Issues That Might Hamper a Safe Oncofertility Practice?

### 5.1. How Does SARS-CoV-2 Act on the Reproductive System?

The novel SARS-CoV-2 in genomic analysis presents a 50% sequence identity with MERS (Middle East Respiratory Syndrome) and 79% with SARS-CoV. SARS-CoV-2 spike (S) protein use angiotensin-converting enzyme 2 (ACE2) as a receptor to enter the host cell, depending on the expression of transmembrane serine protease 2 (TMPRSS2). TTMPRSS2 dissociate ACE2 receptor favoring the entry of SARS-CoV-2 into the host cell [32,33]. The virus infects the human body through respiratory pathways and enters various cells that express ACE2 receptors, including lung, heart, gastrointestinal tract, kidney, testis, and ovaries, leading to multi-organ affection. Therefore, it may be noticed that a higher expression of ACE2 in cells makes them more susceptible to infection with SARS-CoV-2 [33]. It is important to establish how SARS-CoV-2 infection can affect fertility and determine subsequent implications.

#### 5.1.1. SARS-CoV-2 and Male Fertility

ACE2 receptor is more highly expressed in the male reproductive system than females, which can explain gender differences in the infectious rate and a higher fatality rate in men. High levels of ACE2 receptor were found in the testis, in the spermatogonia, Leydig cells, Sertoli cells, and cells of the seminiferous duct [34,35,36]. ACE2 is also expressed in the prostate, responsible for secreting prostate fluid, the main component of the semen [37].

Inflammatory and immunological responses secondary to COVID-19 can cause direct testicular damage or affect testicular function. Oxidative stress induced by the viral infection via inflammatory responses can affect semen quality, sperm function, and morphology and damage sperm DNA [38].

Previous SARS-CoV had a harmful impact on the male reproductive system. Orchitis caused the destruction of the seminiferous epithelium and the function of Leydig cells, changes from fibrosis to the destruction of germ cells due to hyperthermia, and impaired spermatogenesis caused by steroid treatment. It has been shown that the virus causes infertility and increases the incidence of testicular tumors [39]. It has also been shown to destroy germ cells by decreasing the number of sperm in the seminiferous tubule and increasing the thickness of the basement membrane, changes that can be reversible [40]. Similar findings were not reported for SARS-CoV-2, and further studies are needed to analyze the effect on orchitis determinism [37,41].

Shen et al. revealed that ACE2 expression in testis of infertile men is higher than normal and makes them more vulnerable to SARS-CoV-2. ACE2 expression is related to age, with the highest expression in 30-year-old men and the lowest ratio in 60-year-old men, making young males more likely to have testicular injuries secondary to COVID-19 [42]. In postmortem studies, SARS-CoV-2 was not found in the testis [43].

SARS-CoV-2 infection can affect the central nervous system and impair normal function of the hypothalamic–pituitary–gonadal axis, with dysregulation in the pulsatile release of GnRH, which decrease secretions of FSH and LH and abnormal functioning and development of the Sertoli and Leydig cells, which can lead to infertility and affect pubertal development [37]. There is no sufficient evidence that COVID-19 produces transitory or definitive damages. The overall outcome of SARS-CoV-2 infection is influenced by associated diseases, making the patient more vulnerable to induced infertility [37].

#### 5.1.2. SARS-CoV-2 and Female Fertility

Female reproductive system functions, such as follicular development, steroidogenesis, oocyte maturation, ovulation, endometrial regeneration, and embryo development, are related to ACE2. ACE2 receptors are present in the breasts, endometrium, tubes, vagina, and ovary [35,44,45]. In the ovary, ACE2 receptors are expressed in the oocytes, stroma, and perivascular cells of the ovarian cortex [46,47]. However, the ACE2 receptors and TMPRSS2 are not significantly expressed in the tissues of the female genital tract, making them unlikely to be infected with SARS-CoV-2 [46], but with a theoretical risk of infection [47].

The virus may affect ovarian function oocyte quality with secondary infertility or miscarriage [45]. Herrero et al. have shown that SARS-CoV-2 infection can affect ovarian function and follicular microenvironment, thus affecting reproductive outcomes. Increased IgG antibody titers against SARS-CoV-2 decrease the number of extracted and mature oocytes in patients undergoing ART procedures. It cannot be said whether these changes are reversible or not, as the study was performed for a limited period of 3–9 months post-COVID-19 disease [48]. The presence of viral RNA in ovarian tissue has not been demonstrated [49].

The general recommendation is that prospective parents, ART patients, gamete donors, and gestational carriers who meet the diagnostic criteria for SARS-CoV-2 should avoid becoming pregnant or participate in any fertility programs until the clearance of the infection. Thus, sperm donation, ovarian stimulation procedures, and embryo transfer should be performed in asymptomatic patients in the last 14 days and with a negative COVID-19 test [50].

### 5.2. Oncofertility and COVID-19: A Growing Field with an Unexpected Barrier

Although worldwide fertility societies have recommended suspending elective fertility preservation, cancer patients could still benefit from this opportunity [51,52,53], with the obstacles raised by limitations of treatments under the pandemic state: closure of most fertility units, disruptions in the supply chains, reorganization of hospitals with the consequent postponement of reproductive surgeries, delay of treatment schemes, the reduction of vital interdisciplinary consultations and an uneven character in terms of distribution regarding the most correct and judicious application of the principles that generate medical conduct [54]. There have been cases in which these procedures have been temporarily interrupted in areas severely affected by the pandemic, with increased mortality, as was the case in Italy, in the Lombardy area [55].

Although oncofertility would be an emergency, the closure of non-emergency health services has led to a prolonged blockade of infertility treatment for this category of patients due to the reorientation of resources to other patient categories especially related to COVID-19. Subsequently, the emergence of hybrid hospitals that allowed the treatment of both confirmed cases with COVID-19 and those not infected disrupted the proper conduct of oncofertility services. As a result, there was a decrease in referrals by oncologists of these patients during this period despite recommendations from societies [56].

Indeed, the situation from country to country is extremely heterogeneous regarding the addressability of these patients to medical services, this being evident in developing countries. The experience on FP procedures of an Italian hospital-based tissue establishment during lockdown showed a reduction of 65% in cancer patient addressability, predominantly to the sperm bank. The authors speculate that COVID-19 induced general paralysis of the healthcare system and was associated with a delay in new cancer diagnosis, subsequently diminishing FP demand [57].

In fertility clinics, care practices have been modified to reduce the risk of SARS-CoV-2 infection among cancer patients, a category vulnerable to infection due to immunosuppression. Thus, the number of clinic visits has been reduced, with most consultations being conducted online via telemedicine, which has provided patients with security in terms of infection. COVID-19 risk-screening questionnaires and temperature measurements were implemented in clinics. Additionally, measures of social distance were imposed by limiting the number of people who have simultaneous access to the clinic and only by appointment, banning access to visitors, spacing of chairs 2 m apart, and plexiglass dividers. The presence of medical staff was also reduced, and they were properly equipped with N95 masks and eye protection. The consulting and treatment rooms were thoroughly cleaned between patients [58].

### 5.3. SARS-CoV-2 Testing in Oncofertility Programs

There is typically only one chance for cancer patients to preserve reproductive potential before starting cancer treatment, so a SARS-CoV-2 infection during FP procedures is unacceptable. Universal screening for SARS-CoV-2 infections is important to detect asymptomatic clinical patients who follow fertility treatment [59,60]. All patients seeking ART should make triage, but there is still no agreement on the right method to screen negative patients at triage [61,62]. Periodic testing of health workers in fertility clinics is also needed to minimize nosocomial infection.

Oncofertility patients are typically young and in good health before their malignancy diagnosis, and thus might be predisposed to developing asymptomatic or non-specific symptoms of COVID-19 [63]. A prospective study undertaken during the second half of 2020 in a European fertility unit investigated the seropositivity rates for SARS-CoV-2 antibodies in over 500 triage-negative patients and found that 5.1% of the individuals screened exhibited a positive IgM result. A negative IgM result had a 98% predicted value for denial of infection by the nucleic-acid-amplification tests [64].

At least at the beginning of the FP treatment, screening the patient and partner seems necessary to avoid the negative outcomes of an unidentified SARS-CoV-2 infection in a cancer patient [62]. Serology and molecular tests combined are used ideally to increase detection rates [65].

### 5.4. Fertility Preservation in Cancer Patients during the COVID-19 Pandemic

Oncofertility relies upon obtaining and preserving good quality gametes, embryos, or reproductive tissues safely and efficiently, maximizing the chances that future use will result in fertility restoration and pregnancy. The presence of the COVID-19 pandemic in the practice of oncofertility could hamper optimal results when considering the potential effect of the virus on gametes on the safety of cryopreservation of human reproductive tissues and cells [66].

Cancer patients requiring FP may choose for the cryopreservation of embryos, reproductive cells, and tissues depending on patient age, cancer type, and disease prognosis, correlated with cryopreservation safety in the COVID-19 pandemic [67]. For fertility preservation purposes, reproductive cells and tissues are treated with specific cryoprotectants and immersed in liquid nitrogen (LN2) at ultra-low cooling temperatures of −196 °C [68,69,70]. Due to its high protein structure and low water content, the SARS-CoV-2 virus has an increased cryo-resistance, being stable at 4 °C, reaching maximum viability [71]. It is known that due to the presence of cryoprotectants, viruses can survive the freezing and thawing procedures, and hence one of the main concerns surrounding FP was the possibility of SARS-CoV-2 indefinite survival in LN2 and transmission at the moment of fertility restoration [72].

In 2010, Pomeroy et al. published a study about the presence of infectious organisms in the IVF laboratories and the negligible risks of transmission to and between recipients during cryopreservation and storage [72]. This may indicate a minor chance of SARS-CoV-2 presence in the samples.

Potential infectious disease sources, including the SARS-CoV-2 virus, can be considered sperm, oocytes, and embryos [73]. Many available data exist towards SARS-CoV-2 in the seminal plasma fluid of COVID-19 males. Most of the reports do not support the presence of SARS-CoV-2 in the semen or extraprostatic secretion [74,75,76]. Only one study published by Li et al. detected by RT-PCR the presence of SARS-CoV-2 in 16% of semen samples studied [77]. However, studies suggest that sperm from males infected do not contain the virus and that sperm cryopreservation should not be postponed due to the COVID-19 pandemic [43].

Essahib et al. revealed ACE2 and CD147 receptors, especially on the membrane of the epiblast cells, and suggested a potential infection of the human oocytes and pre- and peri-implantation embryos with SARS-CoV-2 [78]. Baragan et al. revealed undetectable viral RNA of SARS-CoV-2 in 16 oocytes examined from women infected with the virus, so there will not be vertical transmission [79]. Unfortunately, there are no other studies of SARS-CoV-2 presence in the oocytes. The virus was not identified in aspirated follicular fluid either [80]. There is no real evidence if embryos can become infected by the SARS-CoV-2 virus, but some studies reveal that blastocysts have virus receptors [73].

SARS-CoV-2 infection can significantly reduce the proportion of high-quality embryos, and suggestions are to postpone the procedure for 3 months after infection to avoid recruiting gametes exposed to the virus [81].

All the above, and the fact that we are confronting a newly emerging virus led to some good laboratory and tissue practice modifications during the COVID-19. The emphasis is on rigorous personal protective equipment for laboratory hygiene, quarantine of samples from known COVID-19 patients, adoption of closed vitrification systems, usage of higher media volumes and multiple washes, and adoption of a cautious approach to the handling and preservation of embryos with a breached zona pellucida [73,82]. The individualization of protocols for reproductive samples reduces the risk of cross-contamination and transmission, with safe long-term storage and efficient recovery [73].

Patients can be encouraged that their gametes are not infected with the SARS-CoV-2 virus and can be safely cryopreserved, but screening for SARS-CoV-2 infection testing is obligatory in cancer patients participating in fertility preservation programs [83]. In addition, even the stimulation protocols have been modified with the use of antagonists and ovulation triggers being preferred. Women followed the protocol at home to reduce access and hospitalizations. Additionally, in the case of males after a telephone triage, they were first approached before the cryopreservation process at the sperm bank [57,58].

The fertile prognosis of young cancer patients was negatively influenced by less surgical training in oncologic surgery and few oncology clinical trials [84]. Reproductive surgery for FP in cancer patients has also been affected following the installation of lockdown, with limited access to laparoscopic FP procedures due to hospital overload or, later, the existence of a hybrid character that required the management of severe COVID-19 cases. Due to the increased risk of aerosols among temporary health workers, laparoscopic interventions have been banned, with mini-laparotomy preferred for ovarian tissue cryopreservation, with the associated risk of exposing cancer patients by prolonging hospitalization [85].

Furthermore, FP procedures were negatively influenced due to patient fear of becoming infected and the uncertainty about the future in terms of cancer evolution and long-term fertility [86,87].

### 5.5. Role of Telemedicine in Oncofertility

Telemedicine—involuntarily adopted mainly in 2020—has proven to be a valuable tool that will most probably be heavily used and perfected in the post-pandemic period. For cancer patients, the possibility of online consultations and counseling was an effective way of reducing hospital and clinic visits and the risk of contracting SARS-CoV-2 [88,89].

A real-life experience of an oncofertility program operating during the period of suspension of all other fertility procedures (March–May 2020) revealed that the outcomes of FP were very similar to those of historical 2019 controls, despite significantly fewer monitoring visits and a “blind” approach in triggering final oocyte maturation in one third of cases. Out of the 29 FP cycles during the study period, one had to be canceled because of symptomatic infection with SARS-CoV-2 but was completed after the patient’s recovery from COVID-19 [90].

Roetker and Velez showed that continuous innovation in telehealth methods, home testing as a screening tool, and online consultation with a fertility expert could be important tools for standard assessment of male fertility patients [89]. Merhi and Zhang reported a novel solution for ovarian stimulation at home in women with reduced ovarian reserves during the COVID-19 pandemic on 22 patients for oocyte retrieval. This represented an easy alternative for minimizing exposure to SARS-CoV-2 infection [91].

### 5.6. Psychosocial Aspects of the COVID-19 and Oncofertility

Fertility treatment postponing or cessation due to the COVID-19 pandemic in cancer patients has supplementary psychological and social effects on cancer diagnosis and treatment [92,93].

Cancer patients have an increased risk of SARS-CoV-2 infection and severe complications because of their immunosuppressed status. They must deal with the fear of disease with the virus during hospital visits for FP. Therefore, they choose to follow primary cancer treatment and give up the wish to have a family after cancer is cured [14]. Self-isolation, social distance, and restrictions influenced by the fear of SARS-CoV-2 infection affect well-being and lead to feelings of distress, boredom, loneliness, uncertainty, frustration, fear of the new, increased anxiety, depression, and suicidal behavior. All these feelings can impair cognitive function and decision-making [93,94].

On the other hand, lockdown helped patients improve physical and mental appearance after cancer therapy, according to a study performed with telephone interviews in adolescents and young-adult cancer patients [95]. During the lockdown, levels of clinical suffering were present in a third of patients, associated with published information in the media about the virus, which leads to weakening confidence in medical institutions. The household’s diminished financial possibilities, correlated with reduced incomes, also contributed to increased clinical suffering [96].

To diminish the psychological and psychosocial effects of the COVID-19 pandemic, oncofertility specialists should provide appropriate information and psychological support to their patients. Non-essential social activities have been restricted due to the lockdown period with psychological consequences such as generalized fear, community anxiety, hysteria, and panic behavior [93,97].

The COVID-19 pandemic reshaped infertility patient experience. Evidence shows that implantation and early pregnancy rates have remained unchanged despite increased stress and anxiety [98]. Rapid diagnosis of neoplastic patients at high risk of developing sexual, psychological, and psychosocial disorders, along with prompt and personalized intervention, improves the quality of life [92].

## 6. Discussion

Multiple legitimate questions and concerns have arisen for cancer patients and their healthcare providers during the successive waves of the COVID-19 pandemic. Cancer patients are often unaware that the life-preserving treatments they will undergo can also threaten their future fertility potential. The impact of cancer or its treatment on fertility potential should be addressed as part of the initial counseling for eligible cancer patients. Oncofertility multidisciplinary teams must manage possible side effects secondary to cancer treatment, such as primary ovarian failure, amenorrhea, fertility preservation, possible obstetric complications, and psychological effects [99,100,101].

Moreover, the COVID-19 crisis has affected the medical system by reorganizing the structure of hospitals and focusing on infected patients, thus dramatically affecting cancer care. Overall, all required resources to maintain additional healthcare support must be allocated to offer cancer patients the best standard of care. The oncological and reproductive factors are essential health concerns for many patients, and are associated with social restraint, psychological stress, significant dependence on the public health system due to high costs, and a comprehensive mobilization of material and human resources.

Cancer patients are susceptible to SARS-CoV-2 infection due to biological status, comorbidities, cancer type, and therapeutic management. COVID-19 has made additional impacts on cancer care, including cancer-screening programs and the initiation of treatment [13,29]. Additionally, SARS-CoV-2 infection can also impair an unknown proportion of fertility in these patients. Furthermore, more research is needed to conclude whether these effects are reversible or permanent.

Unlike other medical conditions, fertility screening must be performed over a more extended period for these patients to achieve their goals of improving quality of life and reproduction. FP should be undertaken without compromising cancer care and further increase the risk of SARS-CoV-2 infection.

During lockdown, the access to oncofertility counseling and the initiation of procedures was delayed, reducing the access to FP procedures. Moreover, to diminish the exposure to the virus, treatment initiation was postponed for newly diagnosed patients, or a follow-up appointment was replaced with a telemedicine consultation. In Italy, a reduction of up to 65% in cancer patient addressability to FP procedures was mentioned, with a delay in new cancer diagnosis and reduced FP procedures [57]. Gupta et al. demonstrated the effectiveness of fertility counseling for newly diagnosed cancer patients of reproductive age from 36.7% (June 2019–January 2020) to 70% (February 2021) [102]. Thus, although the number of surgeries for patients of reproductive age with cancer has decreased, the rate of counseling regarding FP has certainly increased.

Universal screening for SARS-CoV-2 infection is important for detecting asymptomatic clinical patients who follow fertility treatment [59,60]. All patients seeking FP clinics should make triage [61,62], and the periodic testing of healthcare workers is needed to decrease nosocomial infection rates [103], even for those vaccinated. The protection measures implemented by the fertility clinics to protect both patients and medical staff divided the staff into shifts, limited staff–patient interaction, sanitized the equipment and implemented strictness regarding the refilling of cryobanks.

The standard of care during a pandemic for male patients is sperm banking with telephone triage [14], and for female patients is simple at-home ovarian stimulation protocols before embryo/oocyte cryopreservation prior to cancer treatment [58,104]. Additionally, operating protocols have been modified by the cessation of laparoscopic interventions due to the risk of transmitting aerosol infection among healthcare workers. Thus, for the cryopreservation of ovarian tissue, mini-laparotomies were preferred even if the duration of hospitalization and the potential risk of SARS-CoV-2 infection of the cancer patient increased [85].

A particularly sensitive issue is the vaccination of cancer patients because the administration of a single dose of vaccine induces an immune response of specific T-cells IFN-γ and/or IL-2 SARS-CoV-2 in 48.2% of patients, and the second dose increases the response to 90.6% (significantly lower than that of the healthy population), because at three months post-vaccine the response decreases faster in patients with cancer. Identifying this vulnerable group by testing requires the need for a post-vaccination booster to protect these patients [105]. Immunization against coronavirus allowed fertility preservation and restoration techniques to gradually resume. This pattern followed the evolution of pandemic waves, despite an increased number of people vaccinated among people with cancer.

Other possible barriers to oncofertility care are the limited resources of developing countries and the lack of FP programs for cancer patients. These have been profoundly affected by disruptions in the healthcare system during the pandemic. Countries where FP is covered by the health system have significantly higher rates of fertility counseling than those without legislation (48.6% vs. 39.6%) [28].

The objective assessment of the impact of COVID-19 on oncofertility must be subject to further clinical studies in the years following the declaration of the SARS-CoV-2 pandemic. COVID-19 has been, is, and will be an additional challenge for cancer care and fertility due to the constant reappearance of new variants.

## 7. Conclusions

Oncofertility must consider the effects of cancer treatment on fertility and develop a strategy tailored to each case. Oncofertility is a program to which more and more countries have joined, being more or less affected by the pandemic despite recommendations of the profiled companies as a medical emergency. This requires a combined effort by oncology services and fertility clinics to restrict the medical system. COVID-19 plays an important role in the evolution of the disease and FP among cancer patients.

Limitations. We selected articles from PubMed and Web of Science Core Collection databases without analyzing articles present in other databases. The review considered only papers published in English and French, which may omit other relevant articles.
